# Extended abstinence from morphine alters sperm smRNA expression and prevents transmission of intergenerational phenotypes

**DOI:** 10.1093/eep/dvaf006

**Published:** 2025-03-20

**Authors:** Dana Zeid, Andre B Toussaint, Carmen Dressler, Angela Harbeck, Reza Karbalaei, Yandrés Cintrón, Andrew Pan, Mathieu Wimmer

**Affiliations:** Department of Psychology & Neuroscience, College of Liberal Arts, Temple University, Philadelphia, PA 19122, United States; Zuckerman Mind, Brain, and Behavior Institute, Columbia University, New York, NY 10027, United States; Department of Psychology & Neuroscience, College of Liberal Arts, Temple University, Philadelphia, PA 19122, United States; Department of Psychology & Neuroscience, College of Liberal Arts, Temple University, Philadelphia, PA 19122, United States; Department of Psychiatry and Behavioral Sciences, Johns Hopkins University, Baltimore, Baltimore 21205, United States; Department of Psychology & Neuroscience, College of Liberal Arts, Temple University, Philadelphia, PA 19122, United States; Department of Psychology & Neuroscience, College of Liberal Arts, Temple University, Philadelphia, PA 19122, United States; Department of Psychology & Neuroscience, College of Liberal Arts, Temple University, Philadelphia, PA 19122, United States

**Keywords:** Epigenetic inheritance, sperm RNA, opioid, intergenerational inheritance, miRNA

## Abstract

Paternal exposure to drugs of abuse can impact addiction-related behaviours in progeny via germline epigenome remodelling. Previously, we found that offspring of morphine-exposed male rats showed increased morphine-taking, diminished adolescent social play, and increased sensitivity to morphine-derived analgesia. Here, we first tested the impact of a 90-day paternal abstinence period following morphine self-administration on the transmission of the aforementioned phenotypes. The previously observed changes in morphine-related behaviours were no longer present in offspring of morphine-abstinent sires. We next compared small RNA (smRNA) content in sperm collected from four sire intravenous self-administration groups: morphine, saline, abstinent morphine, and abstinent saline. Two smRNAs (rno-miR-150-5p and an snoRNA annotated to *Snora42*/*Noc3l*) were differentially expressed specifically between morphine- and saline-treated sperm. No differential expression between abstinent morphine and saline sperm was observed. These data begin to delineate the temporal limits of heritable germline modifications associated with morphine exposure, in addition to identifying F0 germline factors coinciding with the manifestation of F1 multigenerational phenotypes. Furthermore, these data suggest that paternal abstinence at conception can prevent inheritance of germline factors that may alter offspring susceptibility to addiction-related endophenotypes.

## Introduction

Intergenerational inheritance is a phenomenon whereby exposures and experiences occurring during the parental (F0) generation’s lifetime may impact the expression of offspring (F1 generation) phenotypes, independently of inherited genomic DNA and parent–child interactions. Within the past ∼15 years, numerous environmental insults during the parental lifetime, including stress, diet, chemical, and drug exposures, have been associated with a broad range of altered offspring phenotypes [1–21]. Intergenerational inheritance induced by exposure to addictive drugs is a topic of increasing interest given their pervasive use and misuse among the general population. It is well accepted that neuropsychiatric risk stems from a complex combination of genetic and environmental factors. Expanding literature has reinforced an important role for heritable epigenetic [or otherwise extra-genomic, e.g. small RNAs (smRNAs)] germline factors in vulnerability to substance use disorders. Paternal exposure to psychostimulants, opioids, or cannabis can broadly impact addiction-related behaviours in progeny [9, 15, 19, 20, 22–32], with many studies noting corresponding changes in the F0 sperm epigenome and/or RNA content [9, 15, 23, 28, 33]. Few studies have explored the stability of these changes during periods of abstinence, and the functional mechanisms underlying the transmission of paternal drug exposure remain unclear.

Recent data from cannabis exposure models in animals [34–36] and humans [37, 38], as well as one cocaine mouse model [28], suggest that peri-conception F0 abstinence may mitigate intergenerational transmission of addiction-related phenotypes. However, this remains unexplored with respect to paternal opioid exposure. Under our previously established rat model of morphine-induced intergenerational inheritance, F0 sires continued to receive morphine during the breeding period—a measure taken to mitigate impacts of opioid withdrawal on mating behaviours. F1 offspring of morphine-self-administering sires exhibited increased morphine self-administration, impaired adolescent social play, and altered opioid-induced analgesia [29, 30, 39]. For the current study, we further examine the persistence of these phenotypes in F1 offspring bred from morphine-‘abstinent’ sires.

In the paternal line, drug-induced intergenerational inheritance is thought to be mediated by extra-genomic modifications to F0 sperm that are precipitated by the drug exposure. The precise biological pathways underlying germline inheritance and subsequent modification of F1 phenotypes are unclear, although sperm smRNAs, DNA methylation, and chromatin remodelling have emerged as compelling mechanistic candidates in male-line intergenerational inheritance [40–49]. Increasingly, it is thought that these processes collaborate to mediate intergenerational inheritance, possibly with varying relative contributions to the process [43, 50, 51]. Sperm-derived ncRNAs are favourable mechanistic targets: their transmission to the developing zygote, their necessity for viable embryonic development, and their sensitivity to external perturbations have been consistently demonstrated [41, 44, 45, 49, 52–56]. Sperm ncRNAs have further been found to interact with chromatin and DNA methylation processes related to epigenetic inheritance [43, 51, 57]. Direct manipulation of sperm RNA content at the level of fertilization has proven invaluable for identification of strong causal candidates for transmission of intergenerational phenotypes [54, 55, 58, 59]. In the current study, we compared the impact of either morphine or prolonged abstinence from morphine on smRNA sperm content. The 90-day abstinence period implemented within the current design encompasses the full duration of rat spermatogenesis (∼50–60 days [60]), ensuring that sperm involved in conception of abstinent-sired offspring are never directly exposed to morphine.

## Materials and methods

### Animals and housing

**Figure 1.**
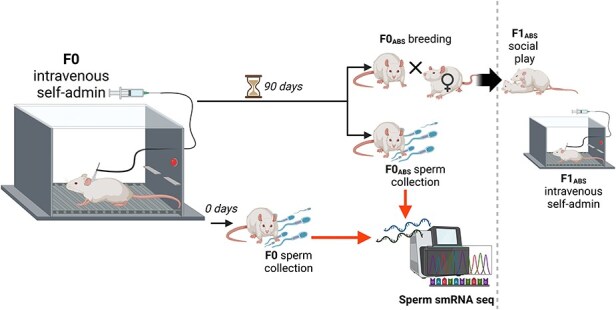
Experimental design depicting the timing of F0 exposure, sperm collection, and breeding to produce the F1 generation. Images generated using ‘BioRender’.

F0 males and females were Sprague–Dawley rats obtained from Taconic Laboratories. Rats were allowed at least 1 week of undisturbed acclimation to the facility prior to beginning experimental procedures. All F1 subjects were bred in-house from F0 breeders. Animals were maintained in a temperature- and humidity-controlled animal care facility on a 12-h/12-h light/dark cycle (lights off at 8:30 am). All behavioural testing was conducted during the lights-off (active) period. Animals were housed in pairs. Food and water were provided ad libitum in home cages. Subjects were handled daily for 2–5 min each for at least 5 days prior to the start of behavioural testing. All procedures were performed in accordance with the NIH Guide for the Care and Use of Laboratory Animals and were approved by the Temple University Institutional Animal Care and Use Committee (record no. 4926). The timeline of F0 self-administration, sperm collection, and F1 breeding are shown in Fig. 1.

### Drugs

Morphine sulphate obtained from Spectrum Chemical (Gardena, CA) was prepared as a solution in sterile 0.9% saline.

### Jugular catheterization surgeries (for morphine intravenous self-administration)

Jugular catheter implantation was performed under ketamine (80 mg/kg)/xylazine (12 mg/kg), IP anaesthesia. An indwelling silastic catheter was threaded subcutaneously over the shoulder blade, inserted in the jugular vein, and sutured in place. The catheter was routed to a mesh back mount platform (Strategic Applications Inc., Lake Villa, IL) and sutured below the skin, between the shoulder blades. Following surgery, catheters were sealed with magnetic caps when not in use and were flushed regularly with 0.2 ml of timentin (0.93 mg/ml, in heparinized saline) to maintain patency. Animals were allowed at least 1 week of undisturbed recovery from surgery before beginning experimental procedures.

### Sire morphine exposure and breeding

F0 males were allowed to lever press for intravenous morphine (daily 3-h sessions, 0.75 mg/kg morphine/59 µl saline, infused over 5 s) or 0.9% saline over 60 days (covering the duration of rodent spermatogenesis [61]). Lever presses were reinforced on a fixed ratio 1 (FR1) schedule, where one lever press resulted in a single infusion of morphine or saline, as described previously [5]. Infusions coincided with a 5-s light cue and were followed by a 20-s timeout period, during which the chamber light was turned off and lever presses were recorded but were not reinforced by infusions. Over 60 days of intravenous self-administration, the average number of infusions per day was 16.10 ± 2.18 (SEM) for morphine-exposed F0 males and 12.02 ± 2.06 (SEM) for saline-exposed F0 males.

A subset of F0 males underwent abstinence for 90 days following the conclusion of intravenous self-administration (F0_ABS_), during which they were housed in the animal facility under normal conditions and handled regularly. After the abstinence period, F0_ABS_ males were each pair-housed with a naïve female over a period of 5 days to breed F1_ABS_ offspring. F0_ABS_ sires were then removed from home cages, and F0 dams were housed individually during the gestation period. F1_ABS_ offspring were weaned at postnatal day (PND) 21 and pair-housed with same-sex littermates.

### F1_ABS_ morphine self-administration

Upon reaching adulthood (PND ∼60), F1 offspring were implanted with silastic catheters, as described earlier. Following recovery, F1_ABS_ offspring were allowed to lever press for intravenous morphine (0.25 mg/kg/infusion) or 0.9% saline in daily 3-h sessions over 10 consecutive days. Lever presses were reinforced on an FR1 schedule, where one lever press resulted in a single infusion of morphine or saline. On day 11, rats were switched to a progressive ratio (PR) schedule, for which the response requirement for each subsequent morphine infusion increased over the course of a 6-h time period.

### F1_ABS_ social play

Social play behaviours were measured in F1_ABS_ aged PND 22–31 (∼early adolescence). The first 2 days consisted of a once-daily habituation phase, during which drug-naïve F1 rats were individually placed in the testing arena (Plexiglas cage filled with cob bedding) for 10 min. Social play testing occurred 24 h after the final habituation session. Prior to beginning the social play trial, each subject was first removed from pair-housing and isolated for 3.5 h. After the isolation period, rats were paired with a play partner (partners were same-sex, with matching sire treatment, and from a different home cage) and video-recorded over 15 min in the testing arena. Pinning and pouncing behaviours were manually tallied from test day videos by two blinded independent scorers. Data averaged across the two scorers were analysed.

### F1 pain assays

Behavioural responses to innocuous and noxious stimuli were analysed at a sub-second scale to identify underlying behavioural features, as previously described [29, 39]. After acclimation to a rectangular plexiglas testing chamber, subjects underwent a set of five stimuli applied to the left or right hind paw. Behavioural responses were recorded at 2000 fps (Photron FastCAM Mini AX 50 170 K-M-32GB—Monochrome). All subjects received all stimuli over 2 days of testing. The order of stimulus presentation was randomized between experimental groups. Videos were scored by blinded experimenters.

### F0 sperm collection

Sperm was collected using a double swim-up procedure from a separate cohort of males exposed to either morphine or saline via intravenous self-administration, as described earlier. Sample collection was timed to match the previously established F0 breeding timepoints (at conclusion of intravenous self-administration for nonabstinent males or 90 days after conclusion of intravenous self-administration for abstinent males). For sperm extractions, rats were first euthanized via live decapitation, and cauda epididymides were immediately dissected and rinsed in phosphate-buffered saline. Cauda epididymides were then transferred to warmed (37°C) embryo culture media (M2 medium with HEPES (Sigma-Aldrich, St. Louis, MO) or Ham’s F-10 medium+GlutaMAX supplement (Gibco, Waltham, MA), and several small cuts were made in the tissue using a scalpel blade to liberate sperm. The tissue and medium were then transferred to a 15-ml conical tube, which was incubated in a water bath at 37°C for 30 min. Following the first incubation, the sperm-saturated supernatant (excluding settled tissue and the layer of media immediately overlying it) was transferred to a clean 15-ml conical tube, which was incubated again in a water bath at 37°C for 10 min. Tubes were then centrifuged for 10 min at ∼6000 g, 4°C to pellet the sperm. The supernatant was then gently poured off, and the sperm pellet was resuspended in a gentle hypotonic solution (0.45% NaCl solution, prepared in nuclease-free water) for an initial wash, then centrifuged again at ∼6000 g (4°C, 5 min) to repellet. The NaCl wash and centrifugation steps were repeated until pellets were visibly clear of bloody contamination (3–4 times for each pellet). The final sperm pellet was flash frozen on dry ice and stored at −80°C.

### Sperm RNA extraction

Sperm pellets were resuspended in nuclear isolation buffer (15 mM Tris-HCL, pH 7.5, 60 mM KCl,15 mM NaCl, 5 mM MgCl_2_, 1 mM CaCl_2_, 250 mM sucrose, 1 mM dithiothreitol) with the following additives (per 10 ml of nuclear isolation buffer): 100 μl of 21 mM AEBSF, 125 μl of 800 mM sodium butyrate, 200 μl of 15% IGEPAL (in nuclease-free water), and one protease-inhibitor tablet (cOmplete Mini EDTA-free Protease Inhibitor Cocktail; Roche, Indianapolis, IN). Resuspended pellets were incubated on ice for 5 min, then transferred to a dry heat bath for 10 min at 37°C. The lysis solution was then transferred to a Dounce homogenizer, and TRIzol LS reagent (Invitrogen, Waltham, MA) was added before homogenization with a tight pestle (∼1 min). The lysate was then moved to a clean centrifuge tube and incubated in a dry heat bath for 5 min at 65°C. Lysate was then transferred back to the Dounce homogenizer, and homogenization with a tight pestle was repeated. The entire lysate was then drawn up and down 5 times through a sterile syringe fitted with a 27-g needle.

After homogenization, the lysate was transferred to a clean centrifuge tube and spun down at ∼800 g for 2 min at room temperature to pellet salts and cellular debris. The resulting supernatant was transferred to a clean centrifuge tube and combined with chloroform by vigorously shaking, then vortexing tubes for over 1 min. Tubes were then allowed to incubate at room temperature for 3 min, after which they were spun down at ∼12 000 g for 15 min at room temperature. The resulting aqueous phase was transferred to a new tube and combined with 2 μl of glycogen (from 20 μg/μl stock) and 600 μl isopropyl alcohol, which was then incubated overnight at −20°C to precipitate RNA.

The following day, samples were spun down at ∼12 000 g for 15 min, 4°C, and the resulting supernatant was pipetted off. Two pellet washes consisting of 75% ice-cold ethanol, followed by centrifugation at ∼8000 g for 6 min, 4°C, were then performed. Ethanol was then carefully pipetted off the washed pellet, which was allowed to air-dry at room temperature. The final RNA pellet was resuspended in nuclease-free water and stored at −80°C.

### smRNA sequencing and analysis

smRNA libraries were prepared from sperm RNA using the NEBNext® Multiplex Small RNA Library Prep Set for Illumina and sent to GeneWiz/Azenta (Burlington, MA) for sequencing as 150-nt paired-end reads on the Illumina HiSeq 4000 platform (San Diego, CA). Analysis was performed using the forward reads. Count matrices and raw sequencing data files are uploaded to the Gene Expression Omnibus (accession no. GSE274906).

Sequencing read quality was assessed using FastQC [62]. Preprocessing and annotation to the miRbase [63] and Ensembl ncRNA rat databases were performed using sRNAbench [64] (see Supplementary Data S1 for sRNAbench settings). Briefly, following adaptor trimming and quality control, sRNAbench aligned reads to the rat genome. This step allows for more accurate annotation of ncRNA transcripts compared to the sometimes used ‘library mapping’ method, which maps directly to an ncRNA database. sRNAbench utilizes a hierarchical mapping approach: reads were first mapped to the miRbase and then filtered out, and the remaining reads were subsequently mapped to the Ensembl ncRNA library.

Annotated miRNA (‘mature_sense.grouped’) and ncRNA (‘ncRNA_sense.grouped’) read files were transferred to RStudio for assembly of count matrices, which were used as input for the DESeq2 differential gene expression analysis pipeline. miRNA and ncRNA matrices were analysed in two separate DESeq2 analyses, for which experimental groups (F0_MOR_, F0_SAL_, F0_ABS-MOR_, and F0_ABS-SAL_) were identified using a combined Abstinence/Treatment factor, allowing for indirect approximation of interaction effects with pairwise comparisons [65]. Differentially expressed transcripts were identified using the following criteria: statistical significance at a Benjamini–Hochberg adjusted *P*-value of <.1 and a log2 expression fold change of >|0.58|.

The DIANA-microT-coding sequence (CDS) algorithm was used to predict targets of rno-miR-150-5p [76] (http://www.microrna.gr/microt_webserver/), with the following settings: species = rat; miRNA resource = MirGeneDB 2.1; interaction score threshold = 0.7; miRNA confidence = high; MRE binding region = all [untranslated region (UTR) and CDS]. qPCR was used to validate differential expression of miR-150-5p, which has a validated assay available as an miRCURY LNA miRNA PCR Assay (Qiagen; Hilden, Germany). cDNA was reverse transcribed from sperm RNA samples using the miRCURY LNA RT kit, and qPCR was run on the Analytik Jena qTOWER3 (Jena, Germany) using the miRCURY LNA SYBR Green Kit. miR-186-5p was used as a housekeeping gene to calculate ΔCT values [66, 67]. ΔCT values were used in the analysis of group differences using unpaired *t*-tests. Data were graphed as 2^−ΔΔCt^ (fold change).

### Statistical analysis of behavioural data

Behavioural data were graphed and analysed using GraphPad Prism (v10.2.2, La Jolla, CA) and ‘jamovi' statistical software (v2.5, the jamovi project, https://www.jamovi.org) using the adapted GAMLj analysis module [68]. Each experimental group included F1 animals sired by four to seven unique sires (see Supplementary Data S2 for the number of sires represented per F1 experimental group).

With the exception of social play, for which each datapoint represented a pair of animals, all analyses included subject ID nested within sire ID as a random factor to account for potential variability associated with litter or sire exposure. If the model failed to converge with this random factor structure (as in the Female Intravenous Self-Administration Days 1–10 and Male Baseline Pain Response analyses), the nested factor was discarded and only subject was retained as a random factor.

Analyses were initially run on female and male F1 data with sex as a fixed factor. Notably, no significant main effects of sex or significant interactions between sex and treatment were identified (data not reported). Females and males were subsequently analysed separately, as described earlier.

Morphine self-administration data were analysed using linear mixed models, with ‘abstinent sire treatment’ and ‘day’ as fixed factors and sire ID as a random factor. PR infusions were compared using Mann–Whitney *U* tests. Baseline pain sensitivity was analysed with a linear mixed model, with ‘abstinent sire treatment’ and ‘stimulus’ as fixed factors and sire ID as a random factor. Pain responses after morphine injection were analysed as linear mixed models, with ‘abstinent sire treatment’, ‘timepoint’, and ‘stimulus’ as fixed factors, and sire ID as a random factor. Finally, social play behaviours (pinning and pouncing) were analysed as separate unpaired *t*-tests, with ‘abstinent sire treatment’ as the independent variable. Nonpatent rats were excluded from intravenous self-administration analyses. Significance was defined as *P* < .05 for all analyses.

## Results

### Morphine self-administration is unaltered in F1 offspring of morphine-abstinent sires

F1 offspring were bred from F0 males that underwent abstinence for 90 days following the conclusion of intravenous morphine or saline self-administration (F0_ABS_). Intravenous morphine self-administration under FR1 and PR schedules of reinforcement was measured in F1 offspring of abstinent sires (F1_ABS_ offspring, see Fig. 1 for the timeline of F0 self-administration, sperm collection, and F1 breeding).

**Figure 2.**
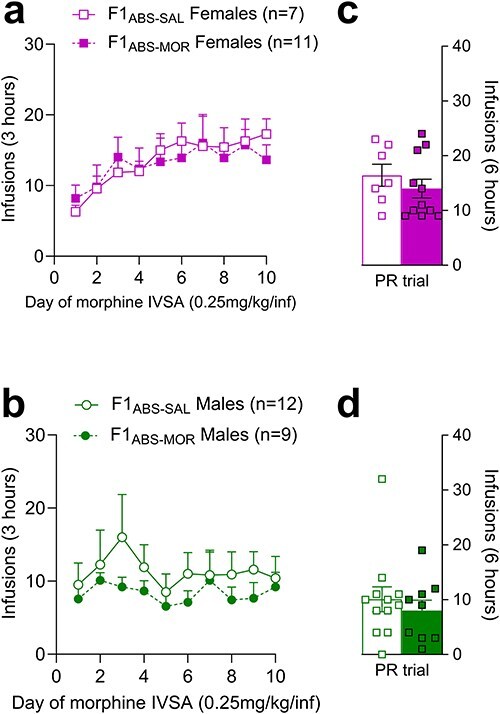
Morphine self-administration at 0.25 mg/kg/infusion over a 10-day acquisition period in F1_ABS_ females (a) and males (b); number of morphine infusions during the PR trial in F1_ABS_ females (c) and males (d).

In F1_ABS_ females, abstinent sire treatment did not impact morphine-taking over the 10-day FR1 acquisition period or infusions under a PR schedule of reinforcement (Fig. 2a, c; Supplementary Data S3). F1 male offspring of morphine-abstinent sires (F1_ABS-MOR_ males) also self-administered morphine at levels comparable to saline-sired controls, both on an FR1 schedule (Fig. 2b) (effect of sire tx: F_(1, 19)_ = 0.75, *P* = .400; sire tx*day interaction: *F*_(9, 171)_ = 0.32, *P* = .966) and a PR schedule of reinforcement (*U* = 47.50, *P *= .466; Fig. 2d). This is in contrast with our previous findings of increased morphine-taking in F1 male offspring bred from nonabstinent morphine-administering fathers (data available as preprint [30]).

### Adolescent social play behaviour is unaltered in F1 offspring of morphine-abstinent sires

**Figure 3.**
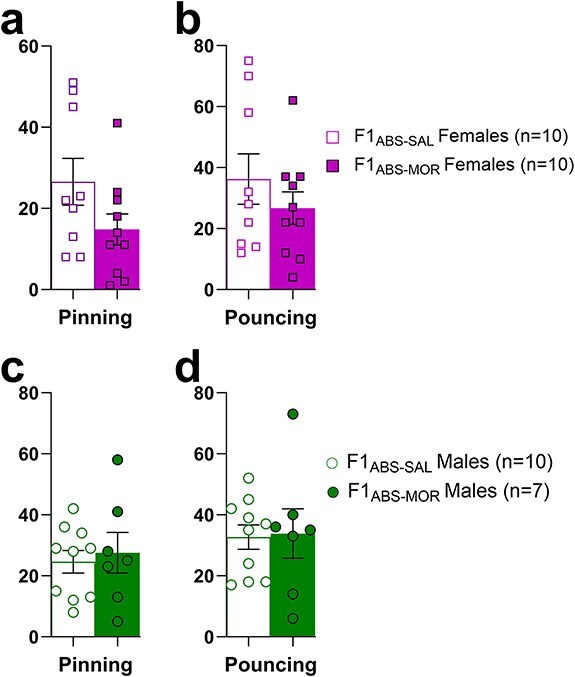
Social play behaviours assessed in F1_ABS-SAL_ and F1_ABS-MOR_ offspring. Top panel: number of F1_ABS_ female pins (a) and pounces (b). Bottom panel: number of F1_ABS_ male pins (a) and pounces (b).

Adolescent (PND 22–31) pinning and pouncing social play behaviours were measured in offspring of morphine-abstinent sires to evaluate developmental phenotypes that may predict susceptibility to opioid addiction in later life. Social play behaviours did not differ by sire treatment in F1_ABS_ females (pinning: *t*_(17)_ = 1.73, *P *= .102, Fig. 3a; pouncing; *t*_(17)_ = 0.99, *P *= .338, Fig. 3b). Similarly, F1_ABS-MOR_ males did not differ in the frequency of pinning (*t*_(15)_ = 0.42, *P *= .68, Fig. 3c) or pouncing (*t*_(15)_ = 0.14, *P *= .89, Fig. 3d) behaviours compared to F1_ABS-SAL_ males. Notably, this contrasts our previous finding of reduced social play behaviours in F1 males bred from nonabstinent morphine-exposed sires [39].

### Baseline pain responses are unaltered in F1 offspring of morphine-abstinent sires

To characterize baseline pain sensitivity in offspring of morphine abstinent sires, behavioural pain responses to five mechanical stimuli were recorded: cotton swab, Von Frey filament at 100 g force (VF100), Von Frey filament at 300 g force (VF300), light pinprick, and heavy pinprick. Height and speed of hind paw withdrawal were scored alongside additional species-typical nocifensive behaviours (eye grimace, paw guarding, paw shaking, jumping, and guarding). The resulting raw data were normalized to *z*-scores, which were then combined into a one-dimensional score using principal components analysis (PCA), as described previously [29, 39]. This transformation produces a PCA-generated pain score that incorporates multiple behavioural dimensions of pain and sets a threshold separating innocuous (‘touch domain’, pain score of <0) from noxious stimuli (‘pain domain’, pain score of >0).

**Figure 4.**
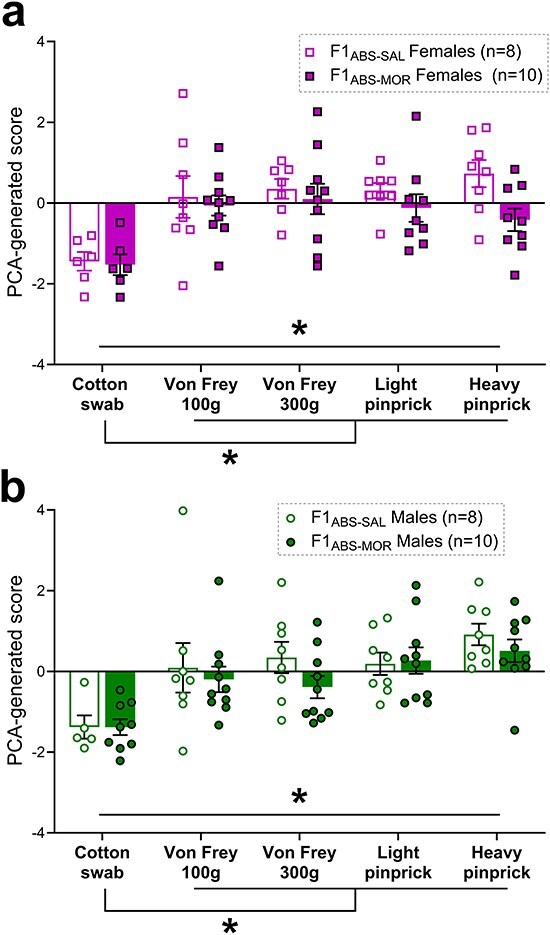
PCA baseline pain scores representing multidimensional behavioural response to five stimuli in F1_ABS_ females (a) and males (b). * = statistically significant at *P* < .05.

Two-way linear mixed models indicated a significant effect of stimulus (stim) but no effect of abstinent sire treatment (tx) in female (stim: *F*_(4,56.1)_ = 12.49, *P* ≤ .0001; tx: *F*_(1,7.9)_ = 1.66, *P* = .234; tx × stim interaction: *F*_(4,56.1)_ = 1.57, *P* = .195; Fig. 4a) or male (stim: *F*_(4,61.5)_ = 10.70, *P* ≤ .0001; tx: *F*_(1,17)_ = 0.86, *P* = .368; tx × stim interaction: *F*_(4,61.5)_ = 0.58, *P* = .676; Fig. 4b) F1_ABS_ animals. In line with our previous use of the PCA-transformed pain score [29, 39], pain responses to cotton swab stimulation registered in the ‘touch-like’ domain (<0) and differed significantly in Dunnett-corrected *post hoc* comparisons from pain scores obtained following VF100, VF300, light pinprick, and heavy pinprick stimulation in both males and females (all comparisons significant at corrected *P *< .001).

### Acute morphine analgesia is not impacted in F1 offspring of morphine-abstinent sires

**Figure 5.**
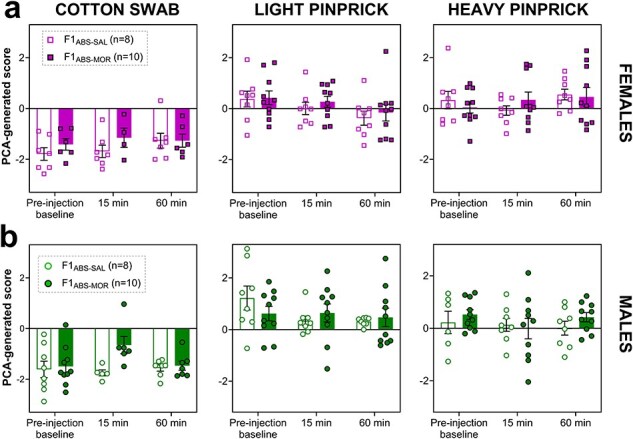
F1_ABS_ female (a) and male (b) PCA pain scores in response to cotton swab (left), light pinprick (middle), and heavy pinprick (right) stimuli at three timepoints (pre-injection baseline, 15 min post morphine injection at 1 mg/kg, and 60 min post morphine injection at 1 mg/kg).

We previously found increased morphine-induced antinociception in male offspring of morphine-exposed sires [29]. To test whether transmission of this phenotype persisted when sires were abstinent from morphine, we measured pain responses to three stimuli (cotton swab, light pinprick, and heavy pinprick) following administration of 1 mg/kg morphine, in F1 offspring of morphine-abstinent sires. Baseline behavioural response to the assigned stimulus was recorded just prior to morphine injection and again 15 and 60 min after the injection timepoint. A three-way mixed model in F1_ABS_ females revealed no significant main effects of timepoint (*F*_(2,111.9)_ = 0.12, *P* = .882; Fig. 5a) or abstinent sire treatment (*F*_(1,8.3)_ = 0.06, *P* = .815), nor any significant interactions with sire treatment (all *P* > .10) on PCA pain scores. However, a significant main effect of stimulus (*F*_(2,112.4)_ = 78.97, *P* ≤ .0001) and a significant interaction between stimulus and timepoint (*F*_(4,111.8)_ = 3.07, *P* = .019) were noted. Holm *post hoc* comparisons revealed a significant difference between pain scores elicited by cotton swab vs. light pinprick and heavy pinprick stimulation (all *P* < .0001) in F1_ABS_ females, although pain scores for light and heavy pinprick did not differ from one another (*P* = .185).

In F1_ABS_ males, no significant main effects of timepoint or abstinent sire treatment were found, nor were any interactions with timepoint or sire treatment noted (all *P* > .10). A significant main effect of stimulus (*F*_(2,116.5)_ = 88.80, *P* ≤ .0001, Fig. 5b) was revealed, and Holm *post hoc* comparisons revealed a significant difference between pain scores in response to cotton swab vs. light pinprick and heavy pinprick stimulation (all *P* < .0001). Furthermore, pain scores differed significantly between light pinprick and heavy pinprick stimulation (*P* = .015) in F1_ABS_ males. Importantly, these results contrast our previous findings in male F1 offspring of nonabstinent morphine-exposed fathers, which exhibited enhanced morphine analgesia [29].

### Differential expression of sperm ncRNAs is exclusive to sperm extracted from nonabstinent F0 males

smRNA sequencing was performed to explore the smRNA signatures of morphine exposure and prolonged abstinence. F0_SAL_ vs. F0_ABS-SAL_ and F0_MOR_ vs. F0_ABS-MOR_ (control ‘timepoint comparisons’) were first assessed to explore potential differences between collection timepoints (at the end of intravenous self-administration vs. 90 days after intravenous self-administration, a difference of age as well as proximity to behavioural testing). The top differentially expressed miRNA in both timepoint comparisons was miR-547-3p, which had increased in expression at the abstinent timepoint. On the other hand, miR-547-3p was not differentially expressed between sperm of morphine- and saline-exposed males in either the abstinent or nonabstinent treatment group comparisons. Three additional miRNAs (miR-126a-3p, miR-200a-5p, and miR-27a-3p) overlapped with the same direction of fold change between the top 10 differentially expressed miRNAs of the two timepoint comparisons, suggesting a common effect of timepoint on sperm miRNA expression (full differentially expressed transcript lists are available at https://doi.org/10.5281/zenodo.14867043). Interestingly, however, a similar pattern was not observed in the Ensembl ncRNA comparisons.

**Figure 6.**
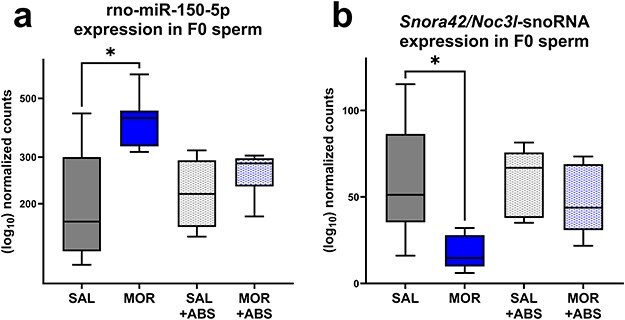
Expression of rno-miR-150-5p (a) and *Snora42/Noc3l*-annotated snoRNA (b) in sperm collected from nonabstinent saline- or morphine-exposed males (F0_SAL_ and F0_MOR_) and males that were 90 days abstinent from saline or morphine (F0_ABS-SAL_ and F0_ABS-MOR_).

Next, we tested the effect of treatment on smRNA content in sperm collected immediately after the conclusion of intravenous self-administration or after a prolonged abstinence period. Between morphine and saline F0 males (F0_MOR_ vs. F0_SAL_), one mature miRNA and one ncRNA transcript were found to be differentially expressed in sperm: respectively, rno-miR-150-5p (Fig. 6a) and a small nucleolar RNA (snoRNA) sequence annotated genomically to an intronic region of the gene *Noc3l* (‘Nucleolar Complex Associated 3 Homolog’), as well as to an snoRNA sequence within the *Snora42/80* family (Fig. 6b) (see https://doi.org/10.5281/zenodo.14867043 for transcript sequences).

In contrast, no miRNA or other ncRNA transcripts were found to be differentially expressed between sperm of morphine- and saline-abstinent F0 males (F0_ABS-MOR_ vs. F0_ABS-SAL_). Notably, both rno-miR-150-5p and the *Snora42/Noc3l*-annotated snoRNA were differentially expressed between F0_MOR_ and F0_ABS-MOR_ but not between F0_SAL_ and F0_ABS-SAL_ (timepoint comparisons), as would be expected if differential expression of these sperm transcripts is specific to the nonabstinence breeding timepoint. Combined with our behavioural data demonstrating that intergenerational phenotypes appearing in F1 offspring bred from nonabstinent males appeared to normalize in F1_ABS_ offspring, this suggests that rno-miR-150-5p and *Snora42/Noc3l* snoRNA are possible candidates for sperm signatures underlying transmission of phenotype from exposed father to offspring. miR-150-5p expression, as measured by qPCR, appeared to replicate the group differences with similar magnitude and statistical significance as sequencing (F0_MOR_ vs. F0_SAL_  *P* = .07; F0_ABS-MOR_ vs. F0_ABS-SAL_  *P *= .42, Supplementary Data S4).

The DIANA-microT-CDS algorithm identified 263 predicted targets of rno-miR-150-5p that met the input criteria. Because these did not include any experimentally validated predictions, we chose to focus only on the top two predicted mRNA transcripts with the highest interaction scores (*Vcsa2* and *Ppp2r1a*), which are reviewed briefly in the Discussion. The full microT-CDS output is available at https://doi.org/10.5281/zenodo.14013319.

## Discussion

Our previous studies demonstrated that paternal morphine exposure led to increased sensitivity to morphine reinforcement and morphine-derived antinociception in adulthood but reduced social play behaviour in adolescence in male offspring [29, 30, 39]. Here, we examined the impact of paternal abstinence following chronic morphine exposure on transmission of intergenerational factors from the F0 to the F1 generation. We found that these intergenerational phenotypes dissipated if F0 sires underwent extended abstinence from morphine prior to mating with drug-naïve females. Apparent disruption in expression of two sperm ncRNAs by morphine was no longer observed in sperm of morphine-abstinent males. In combination, these findings suggest that paternal abstinence surrounding the time of conception may protect progeny from inheriting germline factors that may increase susceptibility to addiction-related behaviours. Overall, these data begin to delineate the temporal boundaries of intergenerational transmission within the lifespan of a living organism, in addition to identifying paternal germline RNAs whose presence coincides with the manifestation of intergenerational phenotypes in offspring.

### smRNA sequencing in F0 sperm

To identify molecules correlated with transmission of morphine-induced intergenerational phenotypes, we compared smRNA content of sperm collected from nonabstinent and abstinent morphine- or saline-exposed adult males. In sperm of nonabstinent morphine-exposed males, two transcripts were found to be differentially abundant between saline- and morphine-self-administering animals: rno-miR-150-5p and an *Snora42/Noc3l*-annotated ncRNA. In contrast, no change in sperm smRNA expression was found in the morphine-abstinent group.

To identify molecules correlated with transmission of morphine-induced intergenerational phenotypes, we compared smRNA content of sperm collected from nonabstinent vs. abstinent morphine- or saline-exposed adult males. In sperm of nonabstinent males only, two transcripts were found to be differentially abundant between saline- and morphine-self-administering animals: rno-miR-150-5p and an *Snora42/Noc3l*-annotated ncRNA. In contrast, no smRNAs were differentially expressed in sperm of abstinent males.

The ‘SNORA’ family of snoRNAs, which includes *Snora42*, canonically guides pseudouridylation of RNAs, a post-transcriptional modification that impacts RNA-mediated cellular processes [69]. snoRNAs and *Noc3l* localize to the nucleolus, snoRNA abundance in the sperm cell varies over stages of spermatogenesis [70], although it is unclear if and how they may be involved in intergenerational inheritance. Henceforth, we direct our discussion to miR-150-5p and its predicted targets, as limited literature exists on this snoRNA outside the realm of oncology research. miR-150-5p is a mature miRNA transcribed from the *Mir150* gene, located on rat chromosome 1. In a rat model of spinal nerve ligation, spinal miR-150-5p was downregulated in rats experiencing mechanical allodynia, and exogenous administration of miR-150-5p lessened this allodynia [71]. Decreased miR-150-5p expression and miR-150-5p knockdown in hippocampus were further associated with increased blood corticosterone and anxiety in mice [72]. Otherwise, this miRNA has not been studied extensively in the context of behaviour and reproduction—most prominently, its expression and functioning have been linked with cancer processes and autoimmune conditions [73–75].

The rat miR-150-5p (rno-miR-150-5p) is further understudied compared to its mouse and human orthologs, which have been associated with several experimentally validated mRNA targets. In contrast, miRNA target databases currently list no experimentally validated mRNA targets of rno-miR-150-5p (likely attributable to the generally scant characterization of the rat genome vs. mouse and human). Fortunately, potential downstream mRNA targets of a given miRNA can be identified using target prediction algorithms, which use conserved seed sequences to predict miRNA–mRNA binding. We used the DIANA-microT-CDS algorithm, which predicts miRNA binding sites in both the 3′-UTR and the CDS, to identify high-confidence predicted targets of rno-miR-150-5p [76]. The top two highest-confidence predicted mRNA targets of rno-miR-150-5p were *Vcsa2* and *Ppp2r1a*. Notably, *Ppp2r1a*, targeted by both the human and mouse miR-150-5p orthologs, and *Smr3a*, a gene with high similarity to *Vcsa2*, are predicted targets of mouse mmu-miR-150-5p.

*Vcsa2* encodes a salivary protein (Smr1-alpha2), which currently has no annotated ortholog in humans or mice, although its sequence is highly similar to other members of the SMR/opiorphin gene cluster that is conserved among rats, mice, and humans [77, 78]. Within this gene family resides *Oprpn*, a relatively understudied salivary opiorphin. The peptide product of *Oprpn* inhibits enkephalin catabolism, and it is thought to act as a peripheral analgesic through this mechanism [79, 80]. Like *Oprpn, Vcsa2* is predicted to be involved in regulation of pain (GO term 0051930 [81, 82]). SMR/opiorphin gene and protein expression is not limited to saliva; indeed, opiorphin is detected in the human bloodstream [83]. Interestingly, *Vcsa2* demonstrates peak expression in the rat testes (Rat BodyMap database, accessed via EMBL-EBI Expression Atlas [84, 85]). Furthermore, SMR/opiorphin gene expression is highly sexually dimorphic and appears to be responsive to endocrine processes underlying stress and reproduction [86, 87]. Thus, *Vcsa2* is a compelling candidate for downstream targets mediating rno-miR-150-5p’s possible regulation of morphine analgesia in offspring of morphine-exposed males.

*Ppp2r1a* encodes a protein phosphatase 2 subunit, an enzyme that has widespread involvement in multiple biological processes. Dysregulation of *Ppp2r1a* is associated with neurodevelopmental and neurodegenerative disorders [88, 89], and it appears to play an important role alongside the alternate structural subunit of protein phosphatase 2, *Ppp2r1b*, in regulation of sperm physiology [90–92]. Thus, both *Ppp2r1a* and *Vcsa2* (expressed in rat testes) may prove informative targets for future characterization of gonadal mechanisms involved in intergenerational transmission. Of course, the involvement of miR-150-5p and *Vcsa2* in pain processes is an interesting parallel to our behavioural data. A significant consideration is that the modulation of pain by morphine “specifically” was impacted in F1_MOR_ animals; thus, any regulation of pain processing via miR-150-5p and downstream targets may only occur in the context of exogenous opioid use.

Finally, rno-miR-547-3p emerged as a potential correlate of timepoint, showing increased sperm expression at 90 days post behavioural testing in both treatment groups. Future studies may aim to investigate this transcript’s possible regulation of aging/time or stress associated with recent behavioural testing.

### Limitations

Some limitations to our methodology should be noted. First, sample size limitations prohibited more direct analysis of interaction effects in smRNA sequencing. Second, this design allowed only for correlation of F0 sperm RNA with F1 behavioural data. Additional work will be required to identify and validate causal factors, as well as their roles in this process. Importantly, the full mechanistic pathways underlying intergenerational inheritance in rodent models—that is, the precise series of events from F0 germline to F1 phenotypes—are still being delineated. Here, we found increased expression of miR-150-5p and an *Snora42/Noc3l*-annotated snoRNA that was specific to the sperm of nonabstinent, morphine-exposed males. miRNAs and other ncRNAs can directly modulate vast downstream expression networks through target silencing [93], although other functions, including complex roles in intergenerational inheritance, have been suggested [43, 94, 95]. For example, sperm ncRNAs could function as first-line environmental ‘sensors’ that proceed to direct translation of exposure signal into more stable epigenetic marks, such as DNA methylation or histone modifications [43, 51, 57, 96]. ncRNAs have diverse origins, and they may be incorporated into the germ cell at several stages of spermatogenesis [44, 97]. This complexity is further embedded within the landscape of hormonal and neurological interactions within the whole organism [97, 98]. Thus, future work should aim to contextualize our findings within a broader mechanistic network.

Our findings should be interpreted with understanding of the limitations associated with this work. Especially as awareness of epigenetic inheritance increases among the general public, it is crucial to emphasize that much more heterogeneity in phenotypic outcomes (and in their effect size) is expected among human populations vs. controlled animal models, given that this phenomenon is driven by environmental factors. Misinformation and harmful rhetoric surrounding epigenetic inheritance have already emerged in popular media, so it is important that we clarify relevance to the everyday person at the time of this publication [99–101]. Our work demonstrates that paternal opioid exposure around the time of conception may increase some addiction-correlated phenotypes among Sprague–Dawley male rats. This does “not” imply that children of human opioid users are fated to addiction. Epigenetically inherited phenotypes should be understood as one among many interacting socioeconomic, biological, and environmental factors that sum to determine whether a given individual crosses the threshold into addictive disorder. As this field develops, we may gain greater understanding of what environmental/genomic variables modulate intergenerational transmission.

## Conclusions

This study explored the temporal dynamics of father/sire-offspring germline transmission in a paternal morphine self-administration paradigm. We found that morphine-taking, adolescent social play, and morphine analgesia phenotypes that were disrupted in F1 offspring of morphine-exposed males were no longer evident in F1 rats bred from sires that were 90 days abstinent from morphine. We further leveraged this design to compare smRNA expression in sperm of males that were nonabstinent vs. 90 days abstinent from morphine, revealing two transcripts with disrupted expression exclusive to nonabstinent male sperm. These findings link morphine-sensitive F0 germline factors to the manifestation of intergenerational phenotypes in F1 offspring, contributing valuable mechanistic insights to the fields of addiction and epigenetic inheritance.

## Supplementary Material

dvaf006_Supp

## Data Availability

Raw sequencing data files are uploaded to the Gene Expression Omnibus (accession no. GSE274906). Additional supplementary data are uploaded to the Zenodo repository at https://doi.org/10.5281/zenodo.14867043.
