# Supplementary material for: Extended abstinence from morphine alters sperm smRNA expression and prevents transmission of intergenerational phenotypes
Source: Environ Epigenet. 2025 Mar 20;11(1):dvaf006. doi: 10.1093/eep/dvaf006 (PMC12097204; doi:10.1093/eep/dvaf006)

S1. sRNABench settings (From "parameters.txt" and "parametersWeb.txt" sRNABench output files)

```
Used genome assembly bowtie1 index: Rnor_6_0_mp
Minimum number of reads: 2
Minimum read length: 15
No maximum read length:


 ---- ANNOTATIONS ----
Used miRNAs reference species from miRBase v22
Used miRNAs for species: rno
Used ncRNA annotations:
   libs=Rnor_6_0_RNAcentral (RNAcentral database 20.0)
   libs=Rnor_6_0_ncRNA (ncRNA from Ensembl release 104 (51 for
Metazoan))
   libs=Rnor_6_0_cdna (cDNA from Ensembl release 104 (51 for
Metazoan))


 ---- Preprocessing and adapter trimming parameters: ----
NEBnext protocol was used with parameters:
   Adapter sequence: AGATCGGAAGAGCACACGTCT
   Minimum length of adapter that needs to be detected: 10
   Allowed mismatches in adapter 1
   Adapter detected starts at position 0 of the read


---- Quality filtering ----
   MEAN quality filtering was used. The read is discarted if the mean
phred score is below the threshold: 20


 ---- Mapping parameters and Annotations ----
Number of allowed mismatches: 1 (mm=1)
Alignment type: seed alignment (alignType=n)
Length of the seed: 20 (seed=20)


Used genome assembly bowtie1 index: Rnor_6_0_mp
Minimum number of reads: 2
Minimum read length: 15
No maximum read length:


 ---- ANNOTATIONS ----
Used miRNAs reference species from miRBase v22
Used miRNAs for species: rno
Used ncRNA annotations:
   libs=Rnor_6_0_RNAcentral (RNAcentral database 20.0)
   libs=Rnor_6_0_ncRNA (ncRNA from Ensembl release 104 (51 for
Metazoan))
```

```
   libs=Rnor_6_0_cdna (cDNA from Ensembl release 104 (51 for
Metazoan))

 ---- Preprocessing and adapter trimming parameters: ----
NEBnext protocol was used with parameters:
   Adapter sequence: AGATCGGAAGAGCACACGTCT
   Minimum length of adapter that needs to be detected: 10
   Allowed mismatches in adapter 1
   Adapter detected starts at position 0 of the read


---- Quality filtering ----
   MEAN quality filtering was used. The read is discarded if the mean
phred score is below the threshold: 20
```

S2. Number of F0 sires represented per F1 experimental groups.

Assay	Experimental group	Sample size	# of unique sires represented
Morphine IVSA	$F1_{ABS-SAL}$ Females	7	4
	$F1_{ABS-MOR}$ Females	11	5
	$F1_{ABS-SAL}$ Males	12	5
	$F1_{ABS-MOR}$ Males	9	5
Social play	$F1_{ABS-SAL}$ Females	10 (pairs)	7
	$F1_{ABS-MOR}$ Females	10 (pairs)	5
	$F1_{ABS-SAL}$ Males	10 (pairs)	7
	$F1_{ABS-MOR}$ Males	7 (pairs)	4
Baseline pain response	$F1_{ABS-SAL}$ Females	8	5
	$F1_{ABS-MOR}$ Females	10	5
	$F1_{ABS-SAL}$ Males	8	5
	$F1_{ABS-MOR}$ Males	10	5
Acute morphine analgesia	$F1_{ABS-SAL}$ Females	8	5
	$F1_{ABS-MOR}$ Females	10	5
	$F1_{ABS-SAL}$ Males	8	5
	$F1_{ABS-MOR}$ Males	10	5
F0 Sperm smRNA seq	$F0_{SAL}$	7	NA
	$F0_{MOR}$	7	
	$F0_{ABS-SAL}$	5	
	$F0_{ABS-MOR}$	6	
F0 Sperm smRNA qPCR	$F0_{SAL}$	7	NA
	$F0_{MOR}$	7	
	$F0_{ABS-SAL}$	6	
	$F0_{ABS-MOR}$	6	

S3. Full statistical analysis results. IVSA = intravenous self-administration, PR = progressive ratio.

	Test	Effect	Figure	df	Test statistic	*p*-value
FEMALES	IVSA morphine infusions, Days 1-10	Sire treatment	2a	1,16	F = 0.02	0.881
		Day	2a	9,144	F = 4.34	<.001*
		Sire tx*Day	2a	9,144	F = 0.45	0.909
	Morphine infusions, PR trial	Sire treatment	1c	NA	U = 47.50	0.466
	Social play	Pinning	3a	17	t = 1.73	0.102
		Pouncing	3b	17	t = 0.99	0.338
	Baseline pain response	Sire treatment	4a	1,16	F = 1.87	0.191
		Stimulus	4a	4,55	F = 12.46	<.0001*
		Sire tx*Stimulus	4a	4,55	F = 1.56	0.198
	Acute morphine analgesia	Sire treatment	5a	1,8.3	F = 0.06	0.815
		Stimulus	5a	2,112.4	F = 78.97	<.0001*
		Timepoint	5a	2,111.9	F = 0.12	0.882
		Sire tx*Stimulus	5a	2,112.4	F = 0.18	0.831
		Sire tx*Timepoint	5a	2,111.9	F = 0.67	0.513
		Stimulus*Timepoint	5a	4,111.8	F = 3.07	0.019*
		Sire tx*Stim*Time	5a	4,111.8	F = 0.45	0.773
MALES	IVSA morphine infusions, Days 1-10	Sire treatment	2b	1,8.12	F = 0.50	0.500
		Day	2b	9,171	F = 0.86	0.564
		Sire tx*Day	2b	9,171	F = 0.32	0.966
	Social play	Pinning	3c	15	t = 0.42	0.680
		Pouncing	3d	15	t = 0.14	0.890
	Baseline pain response	Sire treatment	4b	1,17	F = 0.86	0.368
		Stimulus	4b	4,61.5	F = 10.70	<.0001*
		Sire tx*Stimulus	4b	4,61.5	F = 0.58	0.676
	Acute morphine analgesia	Sire treatment	5b	1,8.1	F = 0.89	0.372
		Stimulus	5b	2,116.5	F = 88.80	<.0001*
		Timepoint	5b	2,116.4	F = 1.16	0.318
		Sire tx*Stimulus	5b	2,116.5	F = 1.15	0.320
		Sire tx*Timepoint	5b	2,116.4	F = 1.34	0.267
		Stimulus*Timepoint	5b	4,115.9	F = 1.06	0.380
		Sire tx*Stim*Time	5b	4,115.9	F = 1.55	0.192

S4. qPCR quantification of mir-150-5p abundance in F0 sperm.

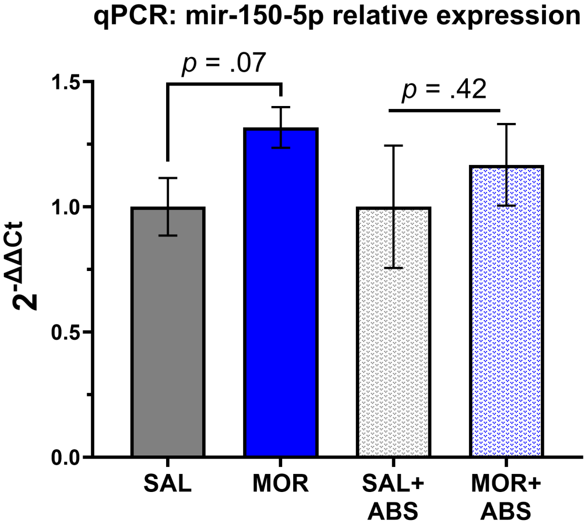

Supplement: dvaf006_Supp [file dvaf006_supp.zip › suppl_data/Zeid et al EE SUPPLEMENT 2_2025.pdf]
